# Dynamic responses to silicon in *Thalasiossira pseudonana* - Identification, characterisation and classification of signature genes and their corresponding protein motifs

**DOI:** 10.1038/s41598-017-04921-0

**Published:** 2017-07-07

**Authors:** Tore Brembu, Matilde Skogen Chauton, Per Winge, Atle M. Bones, Olav Vadstein

**Affiliations:** 1 NTNU Norwegian University of Science and Technology, Departments of Biology, N–7491 Trondheim, Norway; 2 Biotechnology and Food Science, N–7491 Trondheim, Norway; 3 SINTEF Ocean, Brattørkaia 17c, N-7010 Trondheim, Norway

## Abstract

The diatom cell wall, or frustule, is a highly complex, three-dimensional structure consisting of nanopatterned silica as well as proteins and other organic components. While some key components have been identified, knowledge on frustule biosynthesis is still fragmented. The model diatom *Thalassiosira pseudonana* was subjected to silicon (Si) shift-up and shift-down situations. Cellular and molecular signatures, dynamic changes and co-regulated clusters representing the hallmarks of cellular and molecular responses to changing Si availabilities were characterised. Ten new proteins with silaffin-like motifs, two kinases and a novel family of putatively frustule-associated transmembrane proteins induced by Si shift-up with a possible role in frustule biosynthesis were identified. A separate cluster analysis performed on all significantly regulated silaffin-like proteins (SFLPs), as well as silaffin-like motifs, resulted in the classification of silaffins, cingulins and SFLPs into distinct clusters. A majority of the genes in the Si-responsive clusters are highly divergent, but positive selection does not seem to be the driver behind this variability. This study provides a high-resolution map over transcriptional responses to changes in Si availability in *T. pseudonana*. Hallmark Si-responsive genes are identified, characteristic motifs and domains are classified, and taxonomic and evolutionary implications outlined and discussed.

## Introduction

Diatoms are a large and diverse group of unicellular eukaryotic algae. They are crucial components of marine ecosystems, and play an important role in biogeochemical cycling of carbon, nitrogen, phosphorus, silicon and iron. A defining feature of diatoms is the porous, hierarchically nano- and micropatterned, amorphous SiO_2_ (silica) that constitutes the diatom cell wall, or frustule. The low energy cost of silica cell wall synthesis compared with other cell wall types and its protective properties against grazing is believed to be important factors behind the ecological success of diatoms^1–3^.

The diatom cell wall is composed of two major parts: the valves and the girdle bands. These structures are synthesized in separate silica deposition vesicles (SDVs) and exocytosed upon completion^4, 5^. Deposition of Si by diatoms into nano-structured frustules involves many organic components, including proteins like silaffins, cingulins, silacidins, frustulins and pleuralins^6–9^, long-chain polyamines (LCPAs)^7^ and scaffold structures such as the cytoskeleton or chitin fibres^10, 11^. The precursor polypeptides of silaffins, cingulins and silacidins all contain an N-terminal signal peptide for cotranslational import into the ER. Silaffins are subject to extensive post-translational modification, including phosphorylation, methylation, polyamine attachment, glycosylation, sulfation and proteolytic cleavage^12–15^. Polyamine attachment has also been shown for cingulins^16^, whereas silacidins are phosphorylated^9^. Mature peptides from these proteins have *in vitro* silica precipitation activity alone^14, 16^ or in the presence of LCPAs^9, 15^. Once on the outside, the biosilica is enveloped in organic materials, such as polysaccharides, glycoproteins and amino acids, which protect against catabolism and provide the outer surface with active functional groups like carboxyls and amines^17^.

The era of molecular studies has provided new information on uptake kinetics and transport of Si into the cells via trans-membrane silica transporter proteins (SITs)^18, 19^. Si metabolism is coupled to the cell cycle in diatoms, and if Si is unavailable, normal progression through the cell cycle will be arrested in the early G1 phase^20^ or in the biprotoplastic stage immediately following cytokinesis^21^. A recent study indicates that SITs take part in sensing silicic acid levels and regulating the cell cycle and frustule biosynthesis based on this information^22^.

The use of Si in biomineralization has also been reported in plants, radiolarians, choanoflagellates and sponges^23–25^. However, the biomineralization mechanism in diatoms appears to be unique^5^. Many of the proteins found to be involved in silica deposition are not found outside of diatoms; some of the proteins, such as silaffins, appear to be lineage-specific or at least very poorly conserved and rapidly evolving.

In the last thirteen years a large amount of genome and transcriptome data from diatoms have become available^26–32^. Several transcriptome studies on diatom responses to Si starvation or replenishment have been performed, mostly on *Thalassiosira pseudonana*, which have become a model diatom for silica biomineralization and frustule synthesis^33–36^. Recent research has revealed large clusters of genes with expression that is correlated with central Si metabolism genes such as Si transporters or silaffins^35, 36^. Proteomic studies of diatom frustules^16, 37^ and responses to Si replenishment^38^ have provided another layer of information regarding Si metabolism and frustule synthesis.

In this study, we aimed to obtain a detailed overview of the signature responses to silicon availability in *T. pseudonana*, and use this information to identify new putative components of the molecular machinery involved in Si metabolism and biomineralization. To provoke distinct shifts in the Si status of the cells and thus a dynamic change in the gene expression, we performed two different Si perturbation experiments: (1) silicon depletion (shift-down) and (2) silicon replenishment (shift-up). We combined studies of gene expression correlation with taxonomic, sequence, motif, domain and phylogenetic analyses to reveal unknown genes involved in silicon metabolism. Several new gene families with putative roles in Si metabolism and frustule synthesis were identified, including 15 transmembrane proteins with common domain structure that included the frustule protein SiMat7. This study provides a high-resolution gene expression map of the dynamic responses to Si availability, and lays the foundation for detailed functional studies of Si-related processes.

## Results

Two complementary experiments were performed to investigate the dynamics of the silicon-responsive transcriptome of *T. pseudonana*. In the first experiment (Si shift-down), steady-state cultures growing on full nutritional complement (f/2, 37.3 μM) were harvested by centrifugation and resuspended in Si-depleted f/2 medium (0.7 μM). In the second experiment (Si shift-up), Si-depleted (0.7 μM), growth-arrested cultures were replenished with Si to about 300 μM in the medium.

### Physiological responses to Si shifts

**Figure 1:**
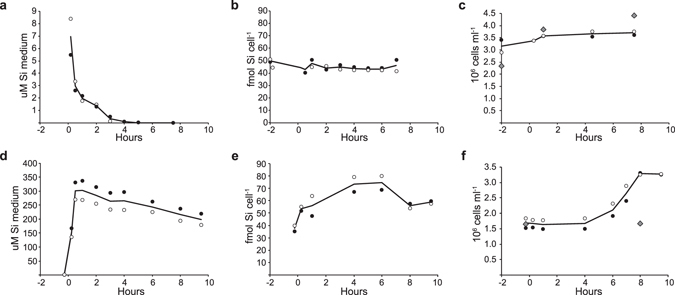
Cell growth and Si content in *T. pseudonana* cultures during silicon shift-down (**a**–**c**) and silicon shift-up (**d**–**f**). (**a**,**d**) Si concentration (μM) in the growth medium. (**b**,**e**) Total particulate Si (fmol cell^−1^). (**c**,**f**) Cell numbers (cells ml^−1^). Two biological replicates (open and closed circles) and average (bold line) are shown. Grey diamonds (**c** and **f**) are cell counts from a control culture.

In the Si shift-down experiment, cells were transferred from a medium with 37.3 (±4.0 STD) μM dissolved Si (dSi) to nearly Si free medium containing 0.7 μM Si, but otherwise a full f/2 nutrient composition. The concentrated cell aliquots contained some residual Si; consequently, the medium in the first sample after transfer contained 7.0 (±2.1 SD) μM dSi (Fig. 1a). The medium Si concentration quickly decreased, and was at or below the detection limit (0.05 μM) after 4 hours (Fig. 1a). Total particulate Si (Si_tot_) was 50.0 (±1.6 SD) fmol cell^−1^ before Si shift-down, and around 45 fmol cell^−1^ during the rest of the experiment (Fig. 1b). Cell numbers varied less than 10% during the experiment (Fig. 1c). The transferred cells came from an exponentially growing population of unsynchronised cells, and some cells were probably close to cell division at the time of transfer. The small increase in cell number within two hours after the transfer to Si-free medium was probably due to cells that had entered the division phase. A control culture grown in Si-replete medium doubled in cell numbers in the same time frame (Fig. 1c).

In the Si shift-up experiment, the concentration of dSi was approximately 300 μM one hour after the Si spike was added to the cultures (Fig. 1d). dSi in the medium decreased with a steady rate for the next 7 hours, and the medium contained approximately 200 μM dSi at the last sampling. Before the Si spike was added, total particulate Si (Si_tot_) was 37.6 (±3.2 SD) fmol cell^−1^, reflecting the Si-deprived status of the cells previous to the experiment (Fig. 1e). After the spike, Si_tot_ increased to a maximum of 74.5 (±8.0 SD) fmol cell^−1^ at t = 6 hours. This increase in Si_tot_ presumably reflects the completion of frustules in dividing cells and replenishment of internal Si pools. Cell numbers gradually increased from 4 hours after Si addition until a doubling was observed after 8 hours, whereas they remained unchanged in a control culture grown without added Si (Fig. 1f). This indicates that the cells were not completely synchronized by Si-limitation prior to the experiment, in accordance with Hildebrand^20^, who showed that 60–80% of *T. pseudonana* cells were arrested in the G1 phase after 24 hours of Si starvation. The doubling time of 8 hours is also consistent with the observations of Hildebrand^20^.

### Transcriptional responses to Si shifts

Global transcriptome analyses of the Si shift-down and Si shift-up treatments were performed, using full-genome oligonucleotide microarrays. Even after strict filtering with regard to statistical significance (p < 0.01), signal strength (spot signal intensity > 200) and expression ratio (log2 expression ratio > ± 1.0 in at least one time point), almost 60% of the *T. pseudonana* transcriptome (6,689 genes) were significantly regulated (see Supplementary Dataset S1). The filtered dataset was subjected to a cluster analysis, and selected clusters of coexpressed genes with interesting expression patterns were investigated further.

The transcriptome dataset was analysed for genes showing a light-dependent expression pattern. The Si shift-up and Si shift-down experiments were performed during the same part of the day; thus, light-dependent expression signatures were expected to be characterised by similar regulation during both experiments (excluding the 72 h timepoint of the Si shift-down experiment). A bioinformatic screen identified 1,461 genes (about 22% of the regulated genes) with such regulation (see Supplementary Fig. S1 and Supplementary Dataset S1). As expected, the light-dependent gene set included genes involved in photosynthesis and the central carbon metabolism. The remaining genes in the dataset were either not regulated by light, or the transcriptional response to Si shift-up or shift-down dominated light-dependent responses.

The Si shift-down and shift-up datasets were compared with the previously published datasets on Si starvation^33, 36^ and Si addback^35^. Of the 15 genes most upregulated after 96 h of Si starvation in the Mock dataset^33^, 13 were also more than two-fold upregulated 72 h after Si shift-down in our dataset (Supplementary Table S1). 2,364 genes were significantly regulated (p < 0.01) both in our dataset, Shrestha^35^ and Smith^36^, and were compared through a cluster analysis (see Supplementary Fig. S2 and Dataset S2). A large fraction of the regulated genes in the datasets of Shrestha^35^ and Smith^36^ showed similar regulation to our dataset (see Supplementary Fig. S2), including the Silaffin-Like Response Gene (SLRG) set^35^. Most of the genes showing opposite regulation to our data in the datasets of Shrestha^35^ and Smith^36^ exhibited a light response-dominated expression pattern in our experiments. In summary, the dataset shows good agreement with previously published studies with regard to Si-responsive genes, and provides high resolution and specificity of the transcriptional Si responses.

### Analysis of Si-responsive gene clusters

The cluster analysis identified three major gene clusters that were characterised by transcriptional suppression by Si shift-down and induction by Si shift-up, suggestive of a role in frustule synthesis: i.e. TPSIL2, CinY1, and SiMat7-like clusters.

1. The silaffin-encoding gene *TPSIL2* was part of a cluster of 20 genes that displayed strongly decreasing expression throughout the Si shift-down time series (5- to 40-fold downregulation, Fig. 2a), and an even stronger induction by Si shift-up, peaking at 4 h after Si addition (30- to 200-fold upregulation, Fig. 2b). The gene expression patterns corresponded closely with those observed by Smith^36^ (Fig. 2c) and Shrestha^35^ (Fig. 2d). Using the SignalP prediction server^39^, 14 of the genes were found to encode proteins with a predicted N-terminal signal peptide (SP) for cotranslational import into the ER, which is indicative of an extracellular localisation. Four genes were also predicted to contain a transmembrane (TM) domain, using the TMHMM prediction server^40^. In addition to *TPSIL2*, this cluster included four genes encoding silaffin-like proteins (SFLPs) identified by Scheffel^41^, three proteins with silaffin-like KXXK-motifs (identified in a screen described below), two proteins containing the extracellular SCP domain (Tp9432 and Tp7900), a trypsin-like serine protease (Tp9434), and a Notch-like EGF repeat protein (Tp24894).

**Figure 2:**
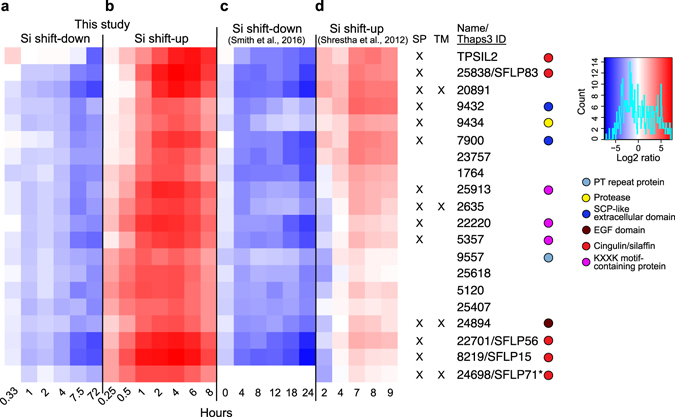
TpSIL2 is part of a cluster of a cluster enriched in Silaffin-like proteins. Heat map representing log2-transformed fold change in expression for the indicated time points of the (**a**) Si shift-down and (**b**) Si shift-up experiments, together with expression data from (**c**) Smith^36^ and (**d**) Shrestha^35^. Numbers indicate JGI Thaps3 gene IDs. The colour key and histogram is shown to the right. Coloured circles indicate the presence of selected known domains. Tp24698/SFLP71 (marked with an asterisk) was not present in the Smith^36^ dataset. SP, signal peptide; TM, transmembrane motif.
2. A larger cluster showed similar, but weaker regulation throughout both experiments (3- to 26-fold downregulation during Si shift-down, 5- to 32-fold upregulation during Si shift-up, see Supplementary Fig. S3). One subcluster displayed two expression peaks at 1 and 4 h after Si addition, whereas the other subcluster mainly peaked at 2–4 h after Si addition. This cluster contained the cingulin-encoding genes *CinY1* and *CinY2* and four *SFLP*s, as well as the frustulin-encoding *FRU1* gene. 37 of the 56 genes in the CinY1 cluster encoded proteins with predicted SP and/or TM domain. The CinY1 cluster contained four genes encoding putative proteases, all with ER signal peptides. Furthermore, three genes in the CinY1 cluster (Tp23433, Tp23434 and Tp2641) encoded DOMON (dopamine β-monoxygenase N-terminal) domain-containing proteins. The *T. pseudonana* genome encodes eight DOMON domain-containing proteins, of which five were induced more than two-fold by Si addition (see Supplementary Fig. S4). Since a DOMON domain-containing protein in insects has been found to protect matrix chitin from chitinase activity^42^, we also investigated the regulation of chitinase-encoding genes. Six genes encoding proteins with a putative chitinase domain were differentially regulated; all of them were down-regulated (see Supplementary Fig. S4).
3. A cluster of 94 genes was tightly coregulated, displaying a narrow expression peak at 2 to 4 h after Si addition (see Supplementary Fig. S5). Close to one third of the genes (32) encoded proteins with predicted SP and/or TM domain. This cluster contained seven genes encoding members of a novel protein family characterised by eight conserved cysteines. Interestingly, one of the family members was identical to SiMat7, a protein detected in the insoluble organic matrix of organic micro-rings and microplates in *T. pseudonana* ^16^; members of the protein family were therefore termed SiMat7-like proteins. This protein family appears to be conserved in diatoms; database searches identified 15 members in *T. pseudonana* and 10 members in *P. tricornutum*. A phylogenetic analysis of the SiMat7-like protein family revealed that it can be divided into four subfamilies that are present in both *T. pseudonana* and *P. tricornutum* (Fig. 3a). All SiMat7-like proteins were predicted to contain an N-terminal ER transit peptide, a canonical protease cleavage site (RXL) and a C-terminal transmembrane domain (Fig. 3b). A comparison of all *T. pseudonana* SiMat7-like family members during the Si shift-up experiment showed a brief down-regulation during the first hour after Si addition followed by an expression peak at 2 h for ten members (Fig. 3c). We analysed a previously published dataset on gene expression throughout a light/dark cycle in *P. tricornutum* ^43^. All SiMat7-like family members in *P. tricornutum* displayed a similar expression profile, with a peak at the end of the light period, which is correlated with cell division-related genes (see Supplementary Fig. S6). The SiMat7-like gene cluster also contained eleven gene products characterised by numerous repeats of a proline-threonine or proline-serine tetrapeptide motif, (XPT/SX), termed proline-threonine (PT) repeats. Three examples of PT repeats are shown in Supplementary Fig. S7.

**Figure 3:**
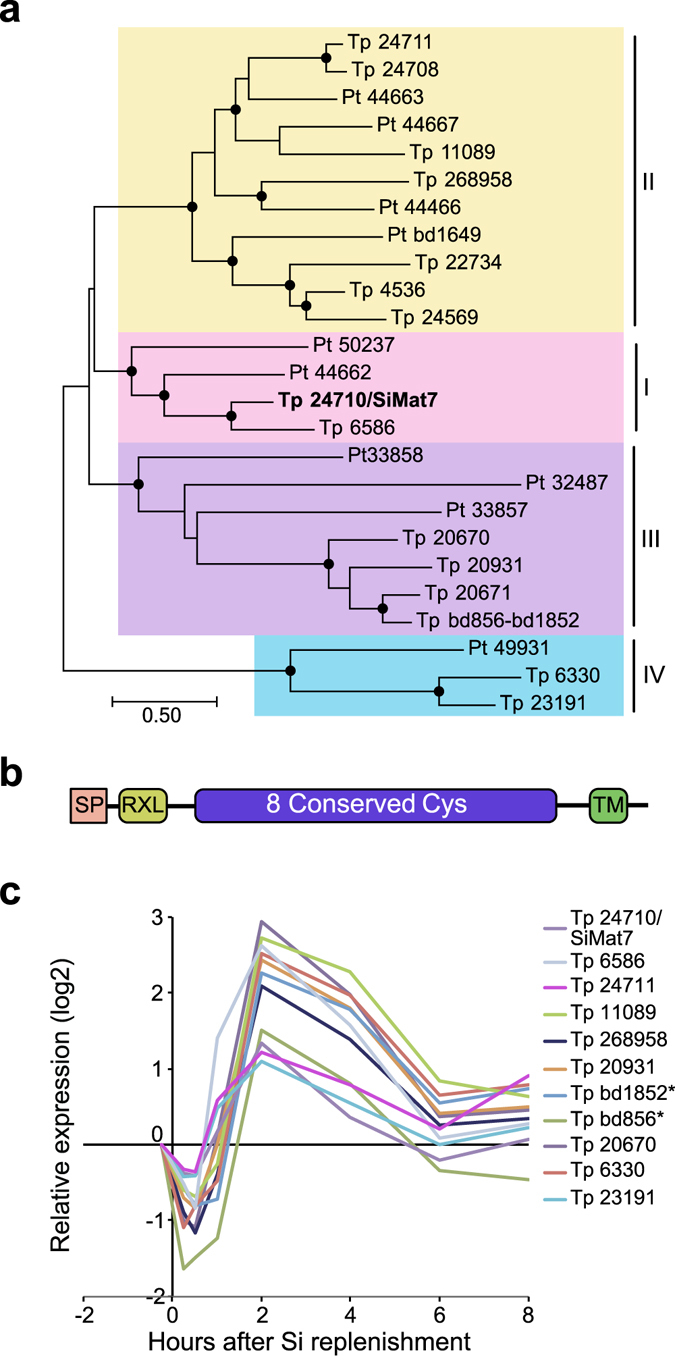
A new family of conserved transmembrane proteins possibly involved in frustule biosynthesis. (**a**) Phylogenetic tree of the SiMat7-like protein family, based on a protein alignment of all SiMat7-like proteins in *T. pseudonana* (Tp) and *P. tricornutum* (Pt). Black circles indicate nodes supported by maximum likelihood and neighbour-joining bootstrap values (1000 × sampling) >80%. The numbers indicate JGI Thaps3 gene IDs. SiMat7 is indicated in bold letters. The SML subfamilies are indicated by colours. (**b**) Predicted domain structure of the SiMat7-like proteins. SP, signal peptide; RXL, protease cleavage motif, TM, transmembrane domain. (**c**) Expression profile of selected *SiMat7-like* genes during Si shift-up experiment. *Tp bd856 and *Tp bd1852* constitute the 5′ and 3′ parts of the same gene, respectively﻿, and not two genes as annotated in the genome assembly.

Since frustule biosynthesis and cell division is closely correlated, we analysed the regulation of 106 cell cycle-related genes (See Supplementary Fig. S8). The expression of most cell cycle-related genes peaked at 1 h to 2 h after Si addition. Four of these genes were also members of the SiMat7-like gene cluster, whereas no cell cycle-related genes were found in the two other Si-responsive gene clusters.

### Silaffin-like proteins can be categorized based on Si responses

As expected, silaffins, cingulins and SFLPs were represented in all the three Si-responsive gene clusters that we investigated in detail. We performed a new screen for silaffin-like proteins, using the presence of an N-terminal ER signal peptide and repeated silaffin-like motifs (KXXK, X = S or G) separated by one to six residues as criterions. The screen identified 10 new KXXK motif-containing proteins, in addition to the SFLPs reported by Scheffel^41^ (see Supplementary Table S2). Among these was Tp25912, which is identical to SiMat4^16^. Poulsen^44^ found that a pentalysine cluster, which is characterised by five non-consecutive lysines within a stretch of 12–14 amino acid residues, was important for silica targeting. The new KXXK motif-containing proteins were screened for this feature, and eight were found to contain one or more pentalysine clusters. Furthermore, all new KXXK motif-containing proteins contained one or more copies of the canonical protease cleavage site. Interestingly, 14 of the SFLPs and KXXK motif-containing proteins, including TPSIL1, also contained PT repeats.

**Figure 4:**
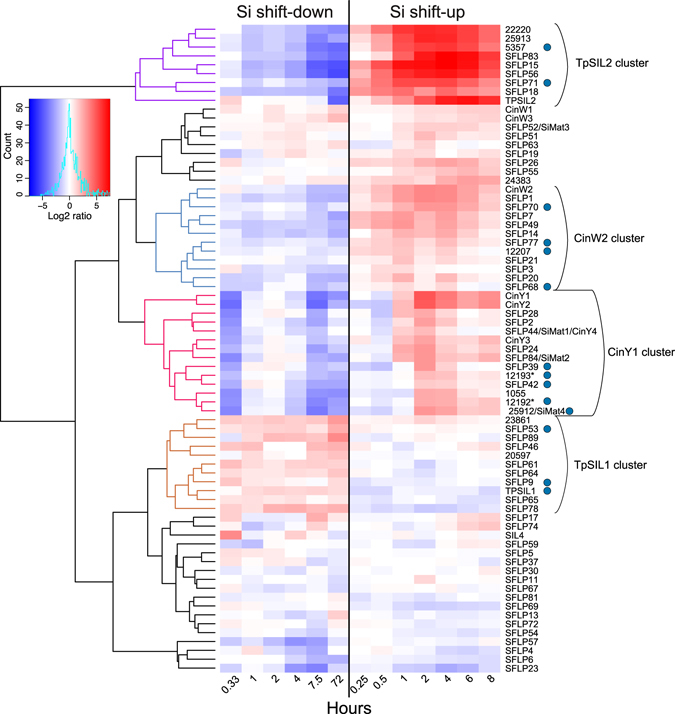
Cluster analysis of *Silaffin-like protein* (*SFLP*) expression during Si shift-up and Si shift-down. Heat map representing log2-transformed fold change in expression for the indicated time points of the two experiments. The colour key and histogram is shown to the left. All differentially expressed *SFLP*s and ten new KXXK motif-containing proteins were included and clustered, discussed clusters are highlighted in colour. *SFLP*s are annotated according to Scheffel *et al*.^41^. Numbers indicate JGI Thaps3 gene IDs. Blue circles indicate the presence of PT repeats. Gene IDs 12192 and 12193 (indicated by asterisks) are likely parts of the same gene.

A cluster analysis was performed on silaffins, cingulins and 55 *SFLP*s significantly regulated by the Si treatments, as well as the 10 new KXXK motif-containing proteins (see Supplementary Dataset S3). The analysis divided the characterised silaffins and cingulins together with SFLPs into distinct clusters of varying size (Fig. 4). Three of the clusters, represented by TpSIL2, CinW2 and CinY1 respectively, displayed down-regulation during Si shift-down and up-regulation during Si shift-up. One cluster, represented by TpSIL1, had an opposite regulation. Other clusters showed weak responses to the treatments, whereas some SFLPs, such as SFLP4 and SFLP6, displayed an expression pattern that appeared to be light-dependent.

### Two highly expressed kinases are co-expressed with SITs

**Figure 5:**
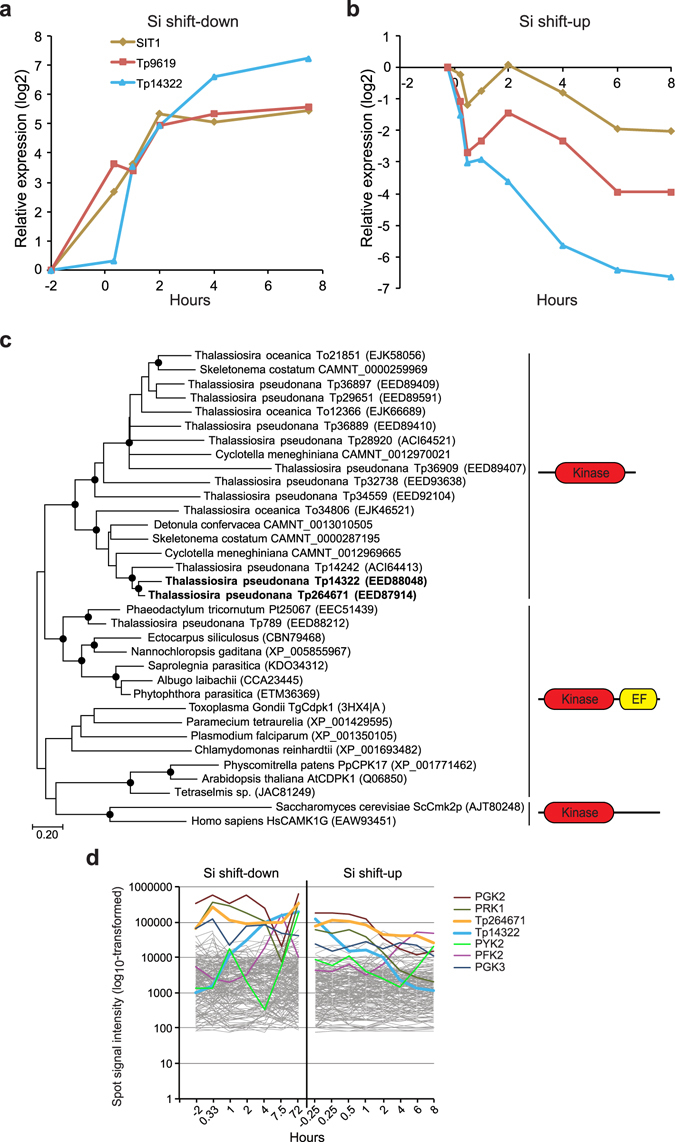
A highly expressed kinase is coexpressed with the silicon transporter SIT1. (**a**–**b**) Expression of the *SIT1* gene cluster represented as log2-transformed fold change during (**a**) Si shift-down and (**b**) Si shift-up. (**c**) Maximum likelihood (ML) and neighbour-joining (N-J) trees were constructed based on a protein alignment of Tp14322 and Tp264671 (shown in bold) and related kinases. The ML tree is shown. The overall topologies for the N-J and ML trees are the same. Black circles indicate nodes supported by ML and NJ bootstrap values (1000 × sampling) >90%. GenBank accession numbers are given in brackets. Domain composition of the different clades is shown to the right. (**d**) Expression levels of differentially expressed kinase-encoding genes during the Si experiments. Expression values are log10 transformed. The seven kinase-encoding genes with highest expression levels are shown in coloured lines, and are listed to the right from top to bottom based on maximum expression level. *Tp14322* and *Tp264671* are shown in bold lines. PFK, phosphofructokinase; PGK, phosphoglycerate kinase; PRK, phosphoribulokinase; PYK, pyruvate kinase.

The silicon transporter *SIT1* was part of a small cluster containing only three highly co-expressed genes that were among the most upregulated in the Si shift-down experiment, except for the first time point (Fig. 5a,b). The other two genes in the cluster, recently also reported by Smith^36^, encoded a kinase (Tp14322) and a diatom-specific protein without any characterised domains (Tp9619). Tp14322 contains no known protein domains besides the kinase domain. Phylogenetic analyses of Tp14322 revealed that it is part of a kinase family related to calcium-dependent protein kinases, consisting of ten members in *T. pseudonana*, that are not found in *P. tricornutum* (Fig. 5c). To investigate the distribution of these kinases in diatoms and other microalgae, tBlastN searches were performed against a database containing 67 transcriptomes from GenBank and the Marine Microbial Eukaryote Transcriptome Sequencing Project (MMETSP)^30^, representing most lineages of marine microalgae and protists (see Supplementary Table S3). Family members were identified in *Skeletonema costatum*, *Cyclotella meneghiniana* and *Detonula confervacea*, which all belong to the Thalassiosirales order, but not in other diatom transcriptomes. A related kinase that is conserved in Stramenopiles, also contains a C-terminal Ca^2+^-binding EF-hand motif; this family is represented by a single gene in *T. pseudonana* (Tp789). Tp14322 is closely related to another kinase in *T. pseudonana* encoded by Tp264671. Interestingly, Tp264671 showed similar regulation to *SIT2* in the Si shift-down experiment (see Supplementary Fig. S9). Furthermore, both Tp14322 and Tp264671 were expressed at very high levels. All significantly regulated genes annotated to encode kinases (169 genes) were compared with regards to their expression levels during both Si experiments (Fig. 5d). Tp14322 and Tp264671 were among the four most highly expressed kinases; the five other kinase-encoding genes showing spot signal intensities above 100,000 all encoded kinases involved in the central carbon metabolism.

### Genes in Si-responsive clusters are not subject to positive selection

**Figure 6:**
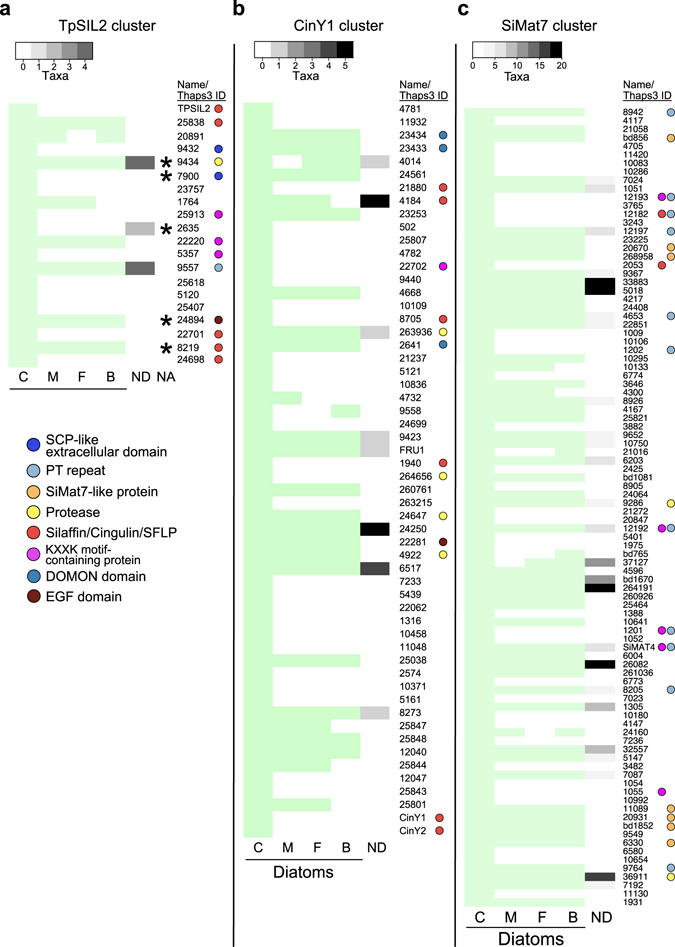
Genes in the Si-responsive gene clusters are poorly conserved. The phylogenetic distribution of proteins included in the (**a**) TpSIL2, (**b**) CinY1 and (**c**) SiMat7 clusters were analysed on a local BLAST server using tBlastN searches. Light green rectangles indicate presence of sequences with significant score against the corresponding *T. pseudonana* protein (tBlastN scores <1e–10) in each diatom class. Greyscale rectangles indicate presence of sequences with significant score in non-diatom microalgae. The greyscale heat map represents the number of species within each taxonomical class with significant score against the corresponding *T. pseudonana* protein. Asterisks indicate significant tBlastN scores (<1e–10) against non-algal sequences in the NCBI nr database. Coloured circles indicate the presence of selected known domains. Numbers indicate JGI Thaps3 gene IDs. C, Coscinodiscophyceae; M, Mediophyceae; F, Fragilariophyceae; B, Bacillariophyceae; ND, non-diatom microalgae; NA, non-algal species.

Many of the genes present in the Si-responsive TPSIL2 (Fig. 2), CinY1 (Supplementary Fig. S3) and SiMat7-like (Supplementary Fig. S5) gene clusters appeared to be poorly conserved, and often lacking similarity to any known domains. The degree of conservation of these gene clusters was therefore investigated. tBlastN searches of each predicted gene product in the clusters were performed against MMETSP transcriptomes, and hits with significant scores (E value < 1e^−10^) were allocated into each of the four diatom classes, and all other microalgae. For 11 of the 20 genes in the TpSIL2 cluster, related transcripts were only found in transcriptomes within the Coscinodiscophyceae class (Table 1, Fig. 6a). Only three of the genes were conserved outside the diatoms. tBLASTn searches of TPSIL2 cluster protein sequences against the non-redundant NCBI database resulted in significant scores against non-algae sequences for five of the genes (Fig. 6a). Similarly, 30 of 56 genes in the CinY1 cluster were significantly conserved only in Coscinodiscophyceae (Table 1). While the cingulins and SFLPs appeared to be restricted to Coscinodiscophyceae, the DOMON domain proteins were found in all diatom classes (Fig. 6b). The SiMat7-like cluster contained less Coscinodiscophyceae-specific genes; still, only 26 of the 94 genes were conserved in non-diatom microalgae (Table 1, Fig. 6c).

**Table 1:** Degree of conservation in selected gene clusters.

Cluster	Genes total	Conserved (# genes/%)	Diatom-specific (# genes/%)	Coscinodiscophyceae-specific (# genes/%)	Positive selection^a^ (# genes/%)
TpSIL2	20	3/15	6/30	11/55	2/10.0
CinY1	56	8/14	18/32	30/54	5/8.9
SiMat7	94	26/28	32/34	36/38	6/6.4

^a^Present in a list of 809 candidates genes of positive selection^45^.

Koester^45^ compared seven strains of *T. pseudonana* to identify candidate genes for positive selection. After rigorous statistical testing, 7% of the protein-coding genes (809 genes) were strong candidates for positive selection. We compared this dataset with the TPSIL2, CinY1 and SiMat7-like gene clusters in order to investigate whether positive selection could be driving the rapid divergence that appears to dominate. As shown in Table 1, between 38 and 55% of the genes in the selected clusters were Coscinodiscophyceae-specific. In contrast, genes identified as candidates for positive selection constituted only between 5 and 10% of the clusters. Thus, when compared with the full genome these gene clusters are not enriched in positively selected genes.

## Discussion

We subjected cultures of *T. pseudonana* to Si starvation and Si replenishment, and performed transcriptome time series analyses to identify putative components of silica biomineralization and their dynamics. Previous studies have analysed *T. pseudonana* responses to Si shift-down^36^ and Si shift-up^35^. These experiments were performed under constant light, whereas the current study was done under a light/dark regime, adding another layer of environmental information. Since both experiments were performed during the same part of the photoperiod, we could identify genes that appeared to be predominantly regulated by the light regime (see Supplementary Fig. S1). Thus, the remaining Si-responsive genes are more likely to have roles specific for Si-related processes. Furthermore, the frequent sampling points during the first hours of both experiments enabled us to capture the dynamics of the initial responses to both treatments, resulting in increased resolution of the gene clusters. Comparison of our dataset indeed showed good agreement for gene clusters with Si-responsive expression in Smith^36^ and Shrestha^35^ (Supplementary Fig. S2, Fig. 2). The differentially expressed gene set also included cell growth-related genes, as diatom cell division is strongly dependent on Si availability^20, 21^. However, the three Si-responsive gene clusters analysed in this study contained few or no genes encoding cell division-associated proteins, suggesting that these gene clusters mostly are involved in other processes (Fig. 2, Supplementary Figs S5, S7 and S8).

Large protein complexes can be assumed to rely on the presence of all its subunits for proper function. While the correlation between transcript and protein abundance is not absolute^46, 47^, components of protein complexes such as the ribosome or the proteasome generally show a high degree of co-expression^43, 48–50^. Frustule synthesis in diatom cultures is strongly correlated with the cell cycle; furthermore, synthesis of the valve and girdle bands is partly separated in time^20, 38, 51^. It is likely that the expression of proteins involved in frustule biosynthesis needs to be coordinated, and that substructures of the frustule need different subsets of proteins. Our cluster analysis of silaffin-like proteins (Fig. 4) revealed four major clusters showing Si-responsive expression, represented by *TpSIL1*, *TpSIL2*, *CinW2* and *CinY2*, respectively. Interestingly, previous localisation studies of silaffins and cingulins^16, 41, 44^ suggest the following distinct localisation for the characterised members: TpSIL1 – valves; TpSIL2 – valves and proximate girdle bands; CinW2 – all girdle bands; CinY2 – distal girdle bands. We speculate that each Si-responsive SFLP cluster is enriched in SFLPs with similar localisation as its corresponding silaffin/cingulin, and thus involved in distinct processes in frustule biosynthesis. The expression pattern of the TpSIL1 cluster, with downregulation during Si-shift-up and up-regulation during Si shift-down, may indicate that TpSIL1 (and other SFLPs in the cluster) are involved in silica deposition under low-Si conditions. In support of this observation, the mature, high molecular isoform of TpSIL1 (tpSil1H) has maximum silica precipitation activity at lower silica concentrations than mature TpSIL2^15^. A majority of the new KXXK motif-containing proteins identified in this study is included in one of the Si-responsive clusters, further supporting a possible role for their gene products in silica deposition.

PT repeats were found in many gene products showing Si-dependent expression, including TpSIL1 and a number of SFLPs and new KXXK motif-containing proteins (Figs 2 and 4, Supplementary Figs S5 and S7). Little is known regarding the function of these repeats. Interestingly, mature tpSil1H peptides were found to contain dihydroxyproline; these residues were suggested to take part in modulation of LCPA-dependent silica formation^15^. Dihydroxyproline has been identified in the cell walls of several diatom species, indicating that this is a general feature of diatoms^52^. Post-translational modification by hydroxylation or dihydroxylation might be important for proper function of other PT repeat-containing proteins as well.

Several lines of evidence suggest a role for the SiMat7-like protein family in diatom cell wall synthesis. Firstly, the expression pattern of most of the SiMat7-like genes is well correlated with *CinY* genes (Fig. 3c, Supplementary Fig. S5). The brief initial transcriptional down-regulation and pronounced expression peak after Si addition probably underscores the importance of proper timing for synthesis and assembly of the frustule. The SiMat7-like genes are also generally expressed at high levels, similar to silaffins and cingulins (see Supplementary Table S1). The domain organization is indicative of a membrane-bound protein with the N-terminal part localized to the non-cytosolic side (Fig. 3b). Also, the SiMat7 protein is associated with organic microrings and microplates^16^; based on the similar domain organization, it is likely that the other family members have a similar localization. It is also interesting that the SiMat7-like proteins are conserved in diatoms, suggesting that these proteins perform fundamental roles during frustule biosynthesis. The expression of all SiMat7-like members in *P. tricornutum* during a day/night cycle^43^ is similar to cell division-related genes, further supporting a conserved function for these genes. The SiMat7-like proteins could be directed to the SDV membrane, and would be attractive candidates to act as transmembrane mediators between cytosolic factors and the SDV lumen. One possibility is that they interact with actin and/or microtubules, likely via cytosolic adapter proteins.

The DOMON domain is found in several secreted or cell surface proteins, and confers copper-dependent monooxygenase activity^53, 54^. Studies of the DOMON domain protein Knickkopf in insects indicate that it is involved in protection of chitin in the newly synthesized cuticle from chitinase activity during molting cycles^42^. *T. pseudonana* frustules contain a chitin-based network organized in a mesh-like scaffold^10^. This network acts as a supporting material for the silica-depositing machinery during frustule biosynthesis. During cytokinesis in plant cells, the polysaccharide callose is believed to provide mechanical support for a tubulovesicular network at the cell plate until cellulose is deposited; at this point, callose is removed, likely by hydrolytic enzymes^55^. If chitin has an analogous role in diatoms, DOMON domain proteins induced during Si shift-up might be involved in protection of chitin network structures from chitinase activity during frustule synthesis.

Seven protease-encoding genes were present in the gene clusters investigated (Fig. 2, Supplementary Figs S3 and S5); of these, six were predicted to contain N-terminal ER signal peptides, suggesting that they may act in the SDV or the extracellular space. These proteases are attractive candidates for processing preproteins of silaffins, cingulins and SFLPs through recognition of their RXL sites to generate the mature peptides^56^. SiMat7-like proteins may also be targets for protease activity, since they also contain RXL sites. Proteases could also be involved in trimming of protein components during frustule maturation.

The silicon transporters SIT1 and SIT2 have been hypothesized to act in monitoring whether environmental silicic acid levels are sufficient to complete cell wall synthesis^22^. The small coexpression cluster (Fig. 4a) including SIT1, a kinase (Tp14322) and an unknown protein (Tp9619) was also identified by Smith and co-authors^36^. They hypothesized that these gene products take part in silicon sensing, possibly activating a transcriptional cascade in response to reduced silicon levels. While we agree that these genes are involved in silicon sensing, the remarkably high expression levels of Tp14322 and the closely related Tp264671 (Fig. 5c) are not indicative of a role in signal transduction cascades, which would be expected to involve low abundance proteins. The substrates of these kinases should rather be expected to be present in large amounts. As no putative ER signal peptides were identified for Tp143232 or Tp264671, they are likely cytosolic, thereby excluding silaffins and other SDV-localized proteins as possible substrates. The SITs could be potential candidates; however, no phosphorylation of SITs has been reported so far. Components of the cell cycle machinery would be attractive targets, but this would necessitate a translocation of the (activated) kinase(s) to the nucleus.

In a recent study of the evolution of the *SIT* gene family in diatoms^57^, *SIT* genes were found to be divided into five distinct clades. Notably, one of these clades (clade E) was found exclusively in members of the Thalassiosirales order. Furthermore, most Thalassiosirales species, including *T. pseudonana*, encoded only clade E SITs. The Thalassiosirales-specific occurrence of the Tp14322 and Tp264671 kinases is interesting in view of these results. These kinases may have co-evolved with the clade E SITs to generate Si-sensing modules enabling species of Thalassiosirales to respond quickly to changing Si levels in the environment.

The tremendous diversity and variability of the diatom frustule on nano-, micro- and mesoscale suggests that the genetic components underlying these structures also are highly diversified: only 57% of the *P. tricornutum* genes are shared with *T. pseudonana* ^28^. The TpSIL2 cluster (Fig. 2) and the CinY1 cluster (Supplementary Fig. S3) described here, with expression patterns indicative of a role in frustule biosynthesis, both contain a majority of genes that are specific to Coscinodiscophyceae (Table 1). Many of these genes are characterised by repeated motifs rather than defined domains, like proteins already shown to be implicated in frustule biosynthesis such as silaffins and cingulins. Even genes encoding proteins with known domains, such as the kinases coexpressed with SITs (Fig. 5), form clades that are phylogenetically restricted.

Two evolutionary mechanisms can explain these results. The genes involved in frustule biosynthesis, especially those encoding structural proteins, could be under very relaxed selection, leading to rapid divergence. This explanation would indicate that many changes in frustule shape and/or patterning are not detrimental to the fitness of the cell. Alternatively, positive selection could drive diversification of frustule biogenesis genes. The switch from a carbon-based to a silicon-based cell wall likely required rapid development of a gene set encoding proteins involved in silicon transport, polyamine biosynthesis and silica deposition. This gene set, or at least parts of it, accumulated major changes in both sequence and copy number during the subsequent diatom diversification. The gene clusters investigated in this study are not enriched in genes listed as candidates of positive selection by Koester *et al*.^45^ (Table 1); thus, relaxed purifying selection appears to be the main evolutionary mechanism driving diversification of these gene clusters. In support of this observation, proteins localized to the extracellular surface of cells interacting with the environment have been found to be under relaxed purifying selection^58, 59^.

## Materials and Methods

### Biological material and general cultivation conditions

*Thalassiosira pseudonana* (CCAP 1085/2) was grown in axenic cultures at 20 °C, illuminated with 130 µmol photons m^−2^ s^−1^ provided by fluorescent tubes (Philips Master TL-D 36 W/840) in light:dark cycles of 16:8 hours. Both experiments described below were initiated at least 4 hours after beginning of the light phase, and all consecutive samplings were performed within the same light period. Cultivation medium was made from natural seawater filtered through 0.2-µm Tuffryn membrane capsules (Pall Gelman) before nutrient amendment and autoclaving. Nutrient amendment was according to the f/2 recipe of Guillard^60^, except that the phosphate (PO_4_^3−^) concentration was increased to 1.25 of the original recipe to prevent limitation at any time during the experiment. Cultures were grown in 2000 mL Nalgene optically clear flasks and aerated to prevent settling of cells and carbon limitation. During the experiments pH was stable in the range 7.5 to 8.0.

Si-free medium was made as follows: A 10 L batch of seawater medium (f/2 without Si addition) was used to grow a culture of *T. pseudonana* until the Si was depleted. The cells were harvested by filtration through a 1 µm filter, and the remaining Si-depleted medium was further filtered through a 0.22 µm filter before it was autoclaved and stored at 4 °C in the dark. The Si-depleted medium contained 0.7 µM Si, which is similar to S_0_ (the concentration of Si where no net uptake occurs^61^) and was used to prepare the experimental medium for the shift-down experiment following the modified f/2 recipe (see above), but with no Si added.

### Si shift-down and Si shift-up experimental conditions

For the shift-down experiment 1000 mL pre-cultures were grown under semi-continuous batch conditions for three weeks prior to the experiment with daily dilutions of approx. 70% of the volume and with surplus of Si. When the cultures were in steady-state, one culture was selected and split into six replicate cultures, and the culture volume of each culture was increased to 1800 mL. Experimental cultures were diluted at the same rate as previously, and Si surplus was confirmed by analysis of Si concentration prior to dilution. The optical density measured at 750 nm (OD_750_) was within the steady-state range for four consecutive days and a Si shift-down situation was established by harvesting the biomass by gentle centrifugation (7500 g, 3 × 10 min). The resulting cell slurry was transferred to Si-free medium (t = 0). Some biomass was lost during centrifugation, and two and two cultures were therefore pooled during the centrifugation. The volume of the slurry re-suspended into the experimental cultures was adjusted so that the OD_750_ in the experimental cultures at t = 0 was equal to the OD_750_ before centrifugation. This was done to ensure that the light regime was the same as before transfer to Si free medium, and thus eliminate light-induced effects on gene expression.

For the shift-up experiment 1000 mL pre-cultures were cultivated as batch cultures in medium with f/4-levels of Si (approximately 55–60 μM Si, including a seawater background of 4 μM) for three weeks prior to the experiment; the cultures were diluted with fresh medium when the Si was depleted. When the cultures were acclimated, one culture was selected and split into three cultures, and culture volume was increased to 1800 mL to have enough volume for experimental sampling. The experimental cultures were kept under the same conditions as previously for four days until the Si was removed from the medium. There was no increase in cell numbers over the next 24 hours. The Si shift-up experiment was initiated by adding a pulse of Si to each culture (t = 0). The new [Si] was approximately 300 μM, which allow at least two cell divisions under unlimited Si conditions before the experiment was terminated.

### Sampling

At each sampling time, two aliquots of approx. 50 mL were removed. One 50 mL aliquot was used to sample RNA, while the other aliquot was used for other analyses: biomass (OD_750_ and cell counts), quantum yield (Q_y_) of photosynthesis to monitor the photophysiological condition of the cells, and Si-measurements - [Si] in the culture medium, intracellular [Si] and total particulate [Si].

Silicon shift-up experimental cultures were sampled right before Si addition, and then every hour or every half hour as the biomass began to increase. Based on the time course of the culture the time points −0.25 (−15 min, control), 0.25, 0.5, 1, 2, 4, 6, and 8 hours relative to the silicate shift-up were selected for further analyses.

The silicon shift-down experiment was sampled before centrifugation (−2 h) and 0.33 hours (20 min) after resuspension in silicate-free medium, and thereafter samples were taken every hour or half hour for 7.5 hours. Samples from time point 72 hours were also included in the microarray analysis, to study if the observed effects of Si depletion lasted longer than an estimated generation time of approximately 8 hours when the cells were kept in Si free medium. The time points −2, 0.33, 1, 2, 4, 7.5, and 72 hours were selected for further analyses.

### Physical and chemical measurements

Cell counts were made with a Bürker hemocytometer on fixed samples (glutaraldehyde, 1–2% final concentration), and at least five counts were made from each sample. OD_750_ was measured to monitor biomass during the experiments, and Qy was measured using a handheld fluorometer (AquaPen-P AP-P 100, Photon Systems Instruments). Nitrate (NO_3_^−^), PO_4_^3−^ and silicate (SiO_4_^4−^) were monitored during the pre-cultivation period and until the experiments started using test kits for seawater samples from Merck and a Merck Spectroquant NOVA60 spectrometer.

To analyse Si concentrations in the medium 5 mL of culture was filtered through GF/F filters and the filtrate was quickly frozen and stored at −20 °C until analysis. Three technical replicate samples of 1.25 mL were pipetted from the filtrate samples and analysed with the molybdate method of Strickland and Parsons^62^. A reagent blank and a standard sample of known [Si] were included in all analytical batches.

Samples for both intracellular Si and total particulate Si consisted of 5 mL of culture filtered onto GF/F filters (two filters for each time point), and the filters were quickly frozen and stored at −20 °C until analysis. One filter was used to analyse intracellular Si after extraction with 4 mL MilliQ water and heating in a water bath at 100 °C for 15 minutes. Three technical replicate samples of 1.25 mL were pipetted from the extract and analysed with the molybdate method. The other filter was used to analyse total particulate Si, and SiO_4_^4−^ was hydrolysed in 4 mL NaOH (0.2N) at 100 °C for 15 minutes. After cooling the extracts were neutralised with 0.35 mL H_2_SO_4_ (1N), and three technical replicate samples of 1.25 mL were pipetted from the extract and analysed with the molybdate method. A reagents blank and a standard sample of known [Si] were included in all analytical batches.

### Isolation of RNA and microarray hybridization

For the RNA isolation and following microarray analysis, 50 ml samples of the biological replicates were quickly harvested through vacuum filtration on Durapore membrane filters (DVPP, 0.65 µm, Merck Millipore). The cells were resuspended in 1 mL of f/2 medium and transferred to 2 mL tubes and centrifuged at 18,000 g for 1 min, and the supernatant was removed. The remaining cell pellet was flash frozen in liquid nitrogen and stored at −80 °C. Total RNA was isolated from the samples using the RNEasy Plant Mini kit (QIAgen). 200 ng RNA from each sample was reverse transcribed and hybridized on a custom-made 8 × 15 K *T. pseudonana* whole-genome 60-mer oligonucleotide microarrays (Agilent Technologies), as described by Chauton *et al*.^43^. Resulting images were processed using Agilent Feature Extraction software version 9.5.

### Statistical analysis

Feature extraction files of the single-color scan data were analysed, and genes with statistically significant differential expression (adjusted p-value < 0.01) were identified using the Limma package (version 3.4)^63^ and R (version 3.0.3). Spots identified as feature outliers were excluded from further analysis, and weak or not detected spots were given reduced weight (0.5). The data were normalized using the quantile method, and no background subtraction was performed. Contrasts between each sampling point and “time zero” (T0) samples (controls) were determined independently for “shift-up” and “shift-down” experiments, and the method of Benjamini & Hochberg^64^ was used to estimate the false discovery rate. Only genes with average adjusted p-value < 0.01 in at least one time point were selected for further analysis. Each gene was represented with one to three corresponding oligonucleotide probes on the microarrays. Genes were annotated based on Thaps3 draft version available from NCBI and supplemented with manual annotations from published articles. The study is MIAME compliant. Microarray data have been deposited in the Gene Expression Omnibus (http://www.ncbi.nlm.nih.gov/geo/) with accession number GSE94329.

### Phylogenetic analyses

Protein alignments were generated using MEGA 7.0.16^65^, followed by manual refinement in GeneDoc 2.7.000^66^. A number of substitution matrices were evaluated (MEGA 7.0.16) and the best one selected. Maximum likelihood (ML) trees were created using the LG model as the amino acid substitution model, with gamma-distributed mutation rates among amino acid sites and partial deletion of gaps and missing data. MEGA 7.0.16 was also used to create bootstrapped neighbour-joining (N-J) trees using the DH model as the amino acid substitution model. A total of 1000 non-parametric bootstrap inferences were executed both for ML and N-J trees.

The phylogenetic distribution of gene products included in selected clusters were analysed on a local BLAST server using tBlastN searches. The database contained assembled cDNA contigs from 60 algae from the Marine Microbial Eukaryote Transcriptome Sequencing Project plus nucleotide and EST data from Bacillariophyta available at NCBI. Transcripts/gene models with blast expect score < 1e–10 were scored as positive and a score matrix produced. Figures and heat maps were produced with the R gplots package v3.0.1 and heatmaps.2.

## Electronic supplementary material

Supplementary figures and tables

Supplementary Dataset 1-3
