# Supplementary material for: Dynamic responses to silicon in Thalasiossira pseudonana - Identification, characterisation and classification of signature genes and their corresponding protein motifs
Source: Sci Rep. 2017 Jul 7;7:4865. doi: 10.1038/s41598-017-04921-0 (PMC5501833; doi:10.1038/s41598-017-04921-0)
Supplement: Supplementary file 1 — Supplementary figures and tables [file 41598_2017_4921_MOESM1_ESM.pdf]

Supplementary Information for

Dynamic responses to silicon in *Thalasiossira pseudonana* - Identification, characterisation and classification of signature genes and their corresponding motifs.

Tore Brembu[1*], Matilde Skogen Chauton[3], Per Winge[1], Atle M. Bones[1] and Olav Vadstein[2]

NTNU Norwegian University of Science and Technology, Departments of Biology[1] and Biotechnology and Food Science[2], N–7491 Trondheim, Norway

SINTEF Fisheries and Aquaculture AS, Brattørkaia 17c, N-7010 Trondheim, Norway[3].

*Author for correspondence:

Tore Brembu

Tel: +47 73 59 62 22 Fax: *+47 73 59 61 00*

Email: tore.brembu@ntnu.no

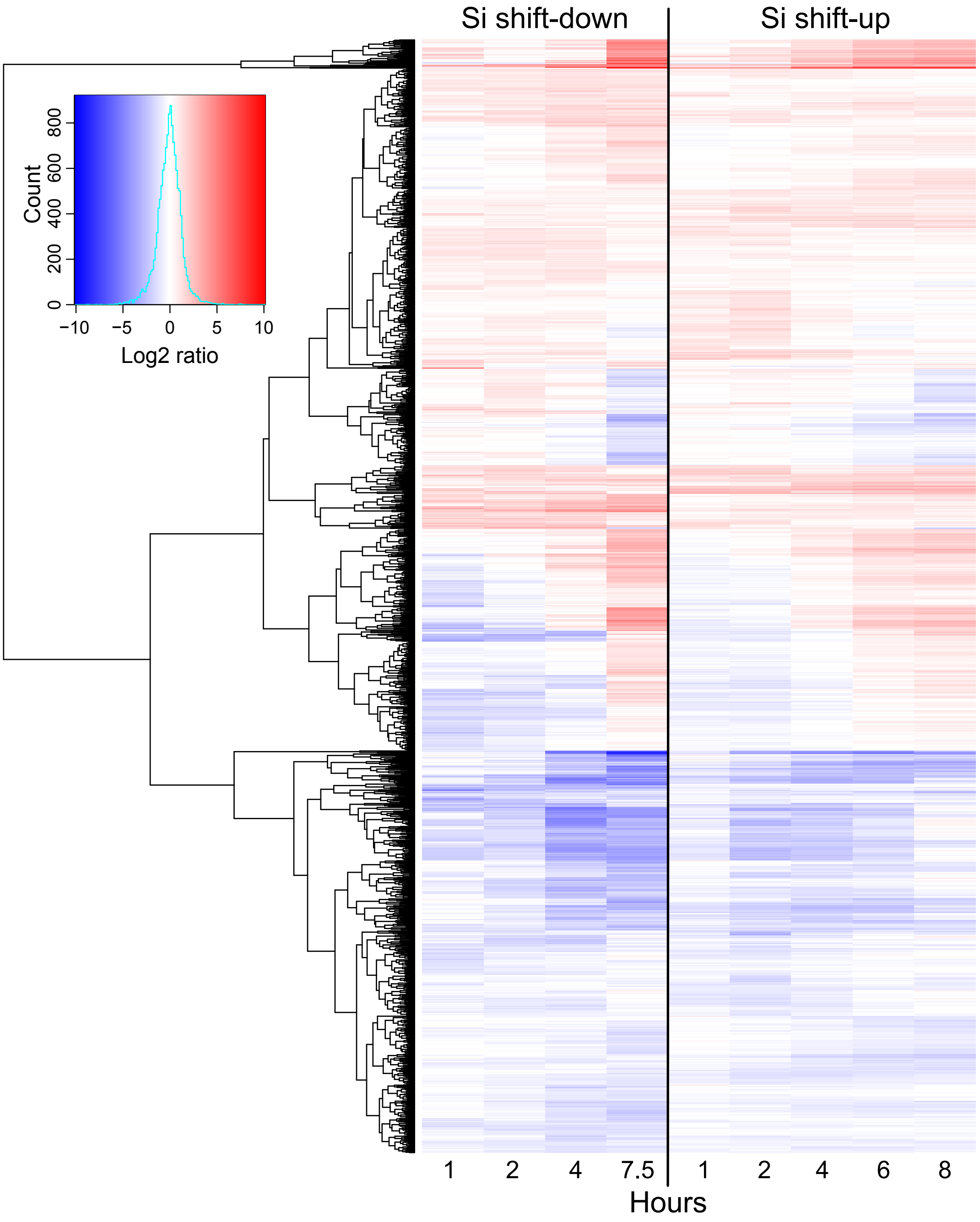

Supplementary Figure S1 Light-regulated genes in the Si shift-down and Si shift-up experiments. Genes with similar expression profiles due to growth under a day-night light regime were identified using the following time points: 1 h, 2 h, 4 h and 7.5 h for the Si shift-down samples, and 1 h, 2 h, 4 h, and the average Log2 value for 6 h and 8 h time point for the Si shift-up samples. Genes that showed a similar type of regulation (up- or down-regulated) at each of the time points in both experiments were identified using a matrix with 16 different expression profiles. Analyses were done with a Perl script and results visualised using the R gplots package (version 2.16.0) and the heatmap2 function. The heat map represents log2-transformed fold change in expression for the indicated time points. The colour key and histogram is shown to the left.

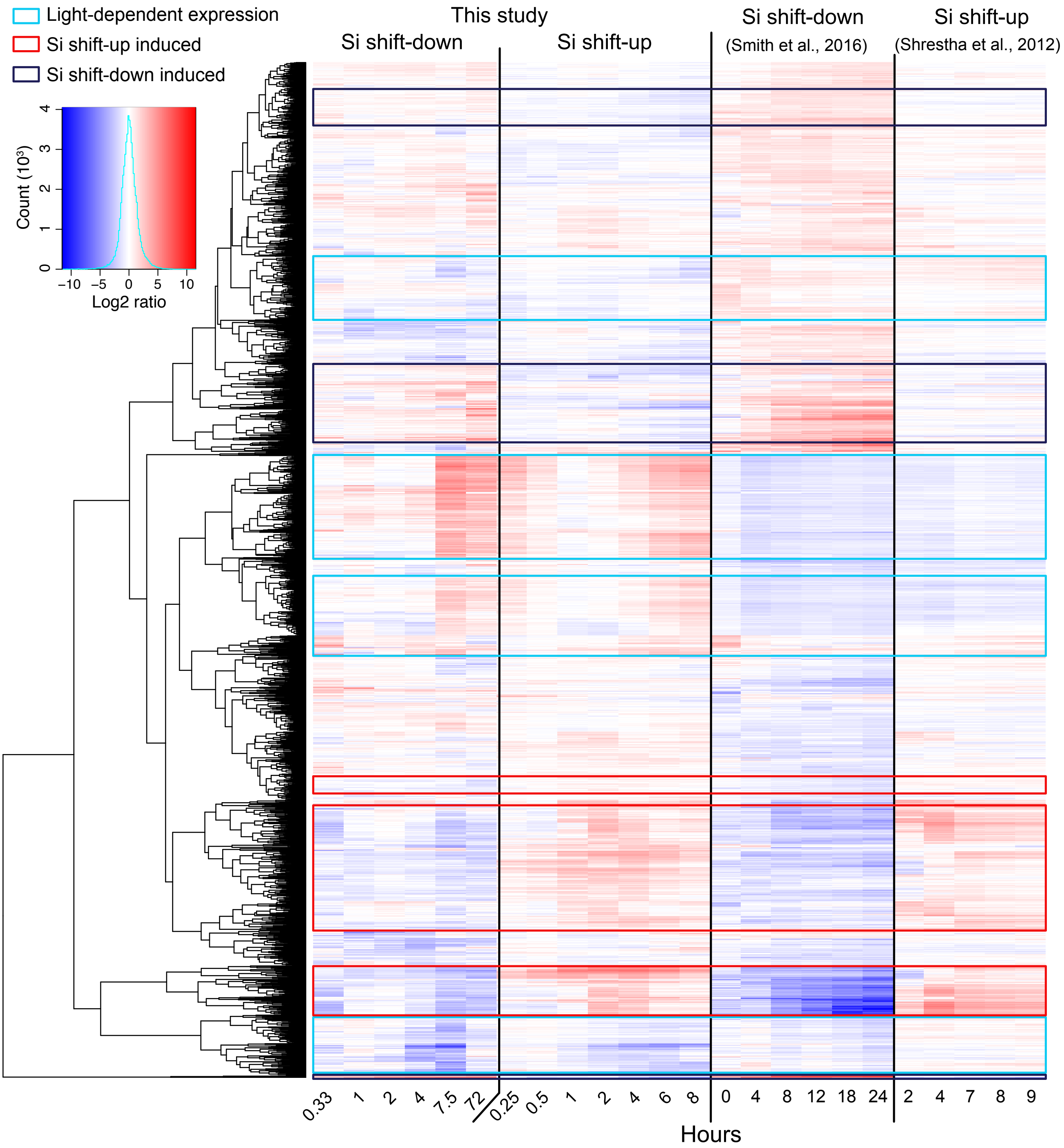

Supplementary Figure S2 Comparison of this study with previously published datasets. A cluster analysis was performed on all genes showing significant regulation in our study, Smith et al. (2016) and Shrestha et al. (2012). The heat map represents log2-transformed fold change in expression for the indicated time points. The colour key and histogram is shown to the left. Gene clusters induced by Si shift-down and Si shift-up are indicated with dark blue and red boxes, respectively. Gene clusters showing light-dependent expression are indicated with light blue boxes.

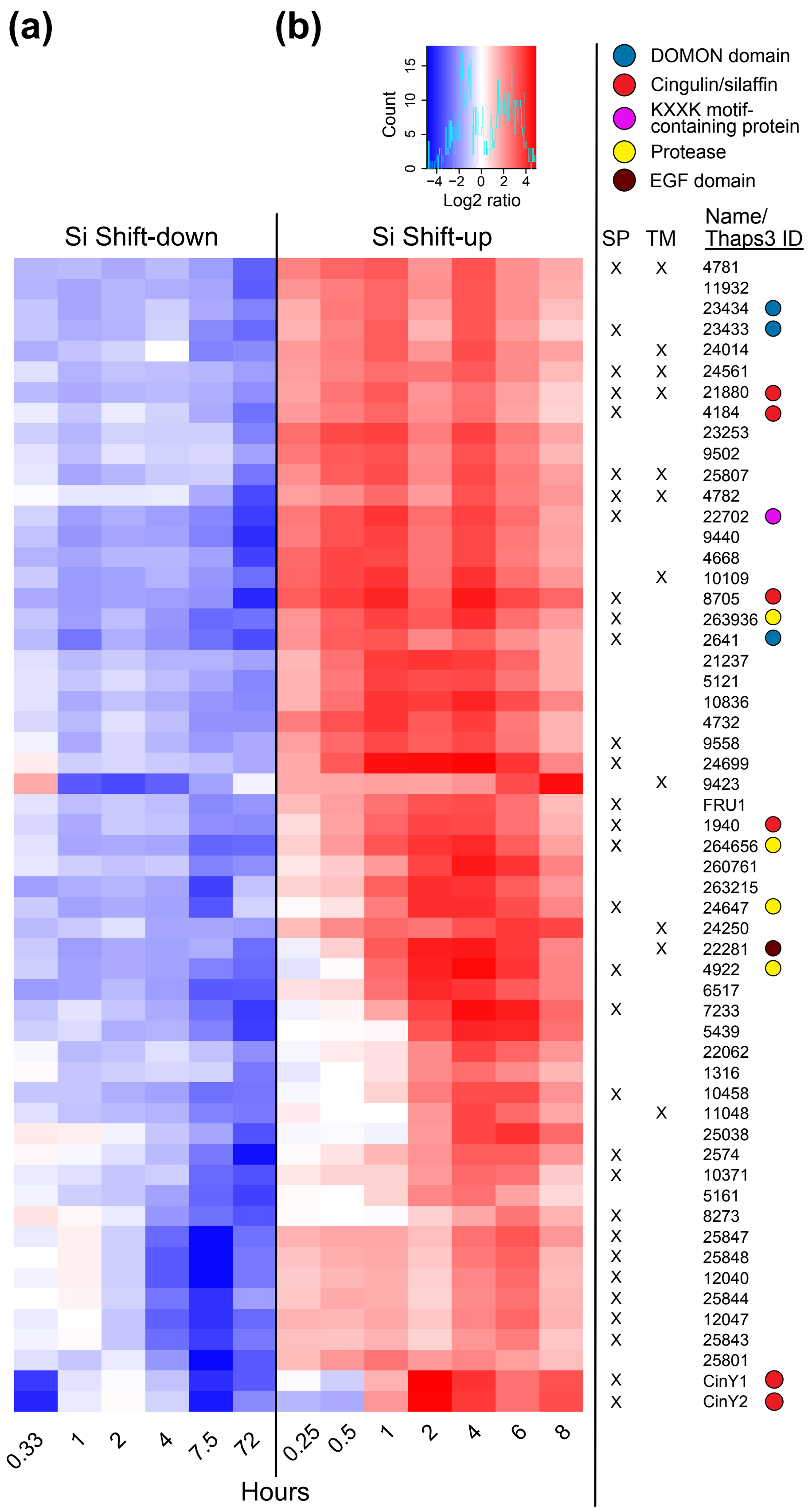

Supplementary Figure S3 Characterisation of the CinY1 gene cluster. Heat map representing log2-transformed fold change in expression for the indicated time points of the (a) Si shift-down and (b) Si shift-up experiments. Numbers indicate JGI Thaps3 gene IDs. The colour key and histogram is shown to the right. Coloured circles indicate the presence of selected known domains. SP, signal peptide; TM, transmembrane motif.

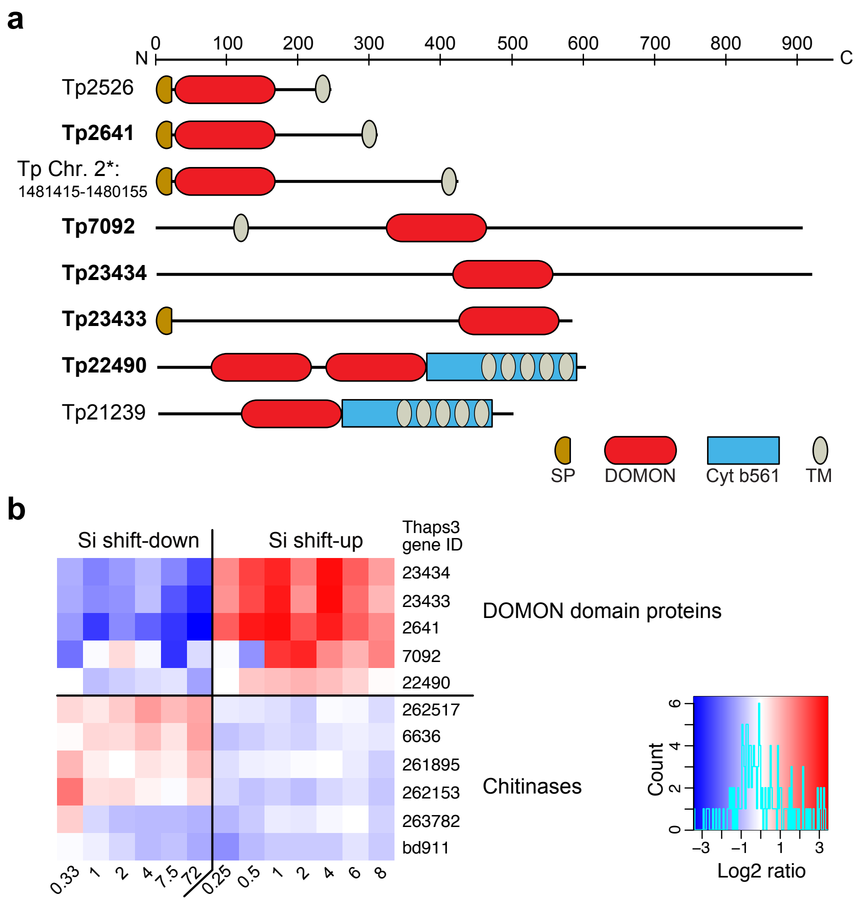

Supplementary Figure S4 DOMON domain proteins in *T. pseudonana*. (a) Domain organisation of DOMON domain proteins identified in *T. pseudonana*. Names written in bold are significantly regulated by Si treatments. A DOMON domain-encoding gene located on chromosome 2 (indicated with an asterisk) was not included in the JGI Thaps3 assembly, but is supported by ESTs. Its sequence coordinates are shown. Cytb561, cytochrome b561; DOMON, dopamine β-monooxygenase N-terminal; SP, signal peptide; TM. transmembrane motif. (b) DOMON-domain protein and chitinase-encoding genes significantly regulated by Si treatments. Heat map representing log2-transformed fold change in expression for the indicated time points of the Si shift-down and Si shift-up experiments. Numbers indicate JGI Thaps3 gene IDs. The colour key and histogram is shown to the right.

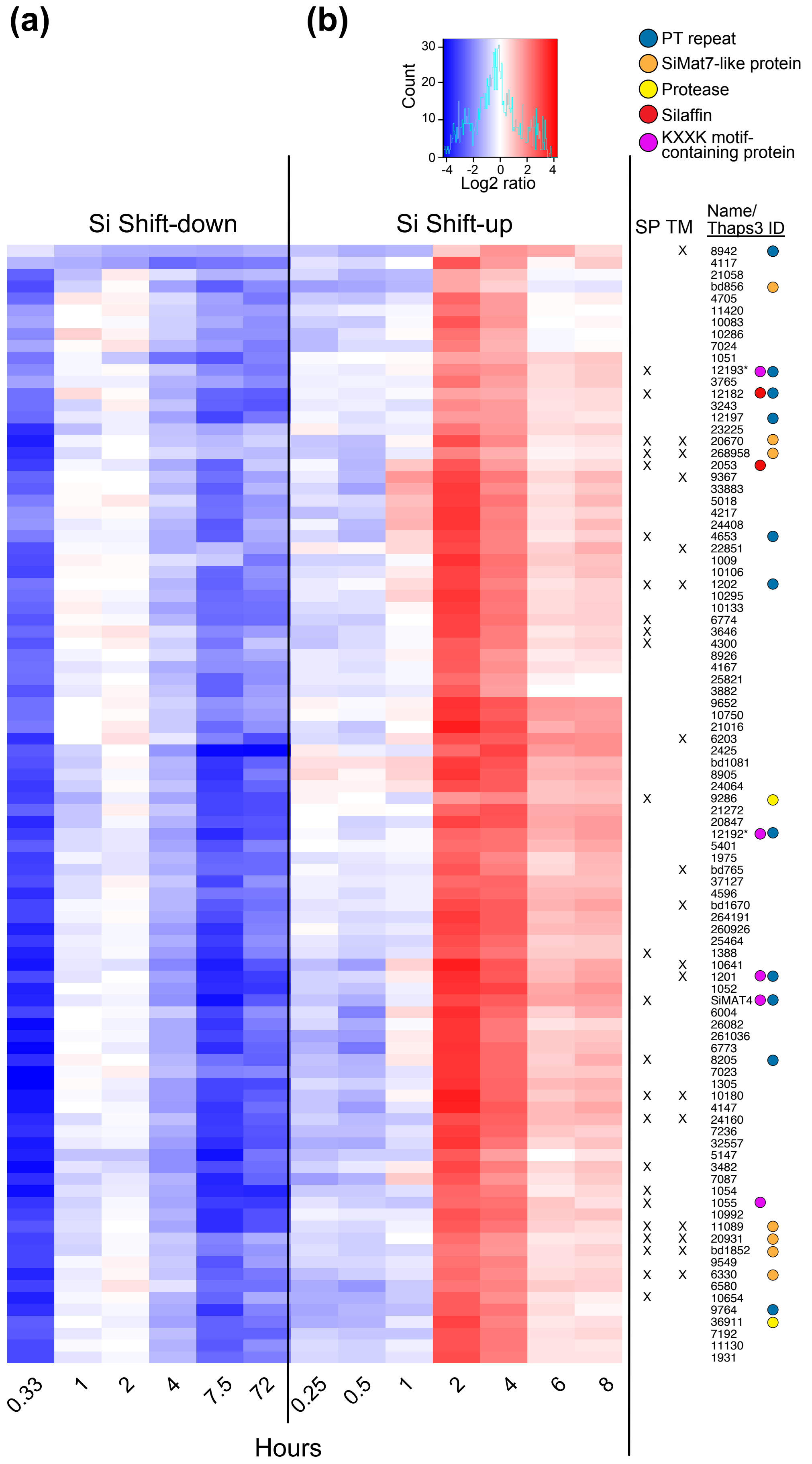

Supplementary Figure S5 Characterisation of the SiMat7 gene cluster. Heat map representing log2-transformed fold change in expression for the indicated time points of the (a) Si shift-down and (b) Si shift-up experiments. Numbers indicate JGI Thaps3 gene IDs. The colour key and histogram is shown to the right. Coloured circles indicate the presence of selected known domains. SP, signal peptide; TM, transmembrane motif.

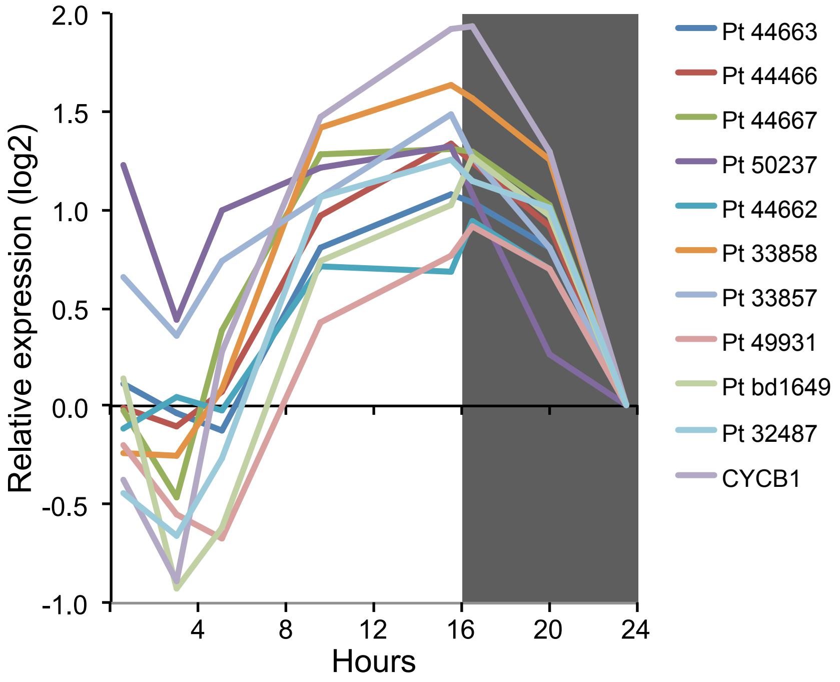

Supplementary Figure S6 SiMat7-like proteins in *Phaeodactylum tricornutum* are coregulated with cell cycle genes over a diel cycle. Expression levels at sampling points (hours after light on) are normalized to the last time point of the dark period (indicated by the dark grey field in the graph). Protein names and Genbank accession numbers: Pt44663 ;XM_002178347, Pt44466; XM_002178513, Pt44667; XM_002178350, Pt50237; (XM_002185163, Pt44662; XM_002178623, Pt3385; XM_002178204, Pt33858: XM_002178204, Pt49931; XM_002184746, Ptbd1649; XM_002176278.1, Pt32487; XM_002177513, PtPtCYCB1; XM_002180361. Data is taken from Chauton *et al.* (2013).

(a)

Tp12192

```
                *         20         *         40
1 : PTLSPTLSPTLSPSLSPTLSPTLSPTLSPTISPSLSPTLS : 18-57
2 : PTVSPTLSPTYA---------------------------- : 65-76
3 : PTNTPTETPSKSPTFFPSWSPTSSPTFVPSFSPTESPILI : 81-120
```

(b)

Tp4653

```
                *         20         *         40         *
1 : PSVAPSDFPTHSPTASPSMSPSVSHM------------------------ :  60-85
2 : PSASPSESPTISTQ------------------------------------ :  86-99
3 : PTSSPSMSPSNSSA------------------------------------ : 100-113
4 : PTSFPSGSPSASPSAFPTSQPSISSM------------------------ : 114-129
5 : PTTTPSMSPSVSSK------------------------------------ : 130-143
6 : PSAFPSASPTDVPSSSPSESPTNFPTTSPTRKPSSVPSSSPSLAPSISSE : 144-203
```

(c)

Tp9558

```
                *         20         *         40
1 : PTLSPTLSPSLSPSQS----------PVWKGDAWK-----ADGY :  72-100
2 : PTLSPTLS----PTEAEFVSPNPTSSPVWKGDAWKTDGWKADGY : 101-140
3 : PTLS--------PTEAEFVSPNPTSSPVWK----------ADGW : 141-166
4 : PTLAPTLSSTESPTDAVFISANPTSSPVWKGDAWK-----ADGY : 179-207
5 : PTLSPTLS----PSDA---------------------------- : 208-219
6 : PTLSPSVS----PTEG---------------------------- : 236-247
```

Supplementary Figure S7 Examples of PT repeats in *T. pseudonana* proteins induced by Si shift-up. (a) Thapsdraft_12192, in the SiMat7-like cluster. (b) Thapsdraft_4653, in the SiMat7-like cluster. (c) Thapsdraft_9558, in the TPSIL2 cluster. The localization of each repeat within the protein sequence in indicated to the right. Numbers indicate JGI Thaps3 gene IDs.

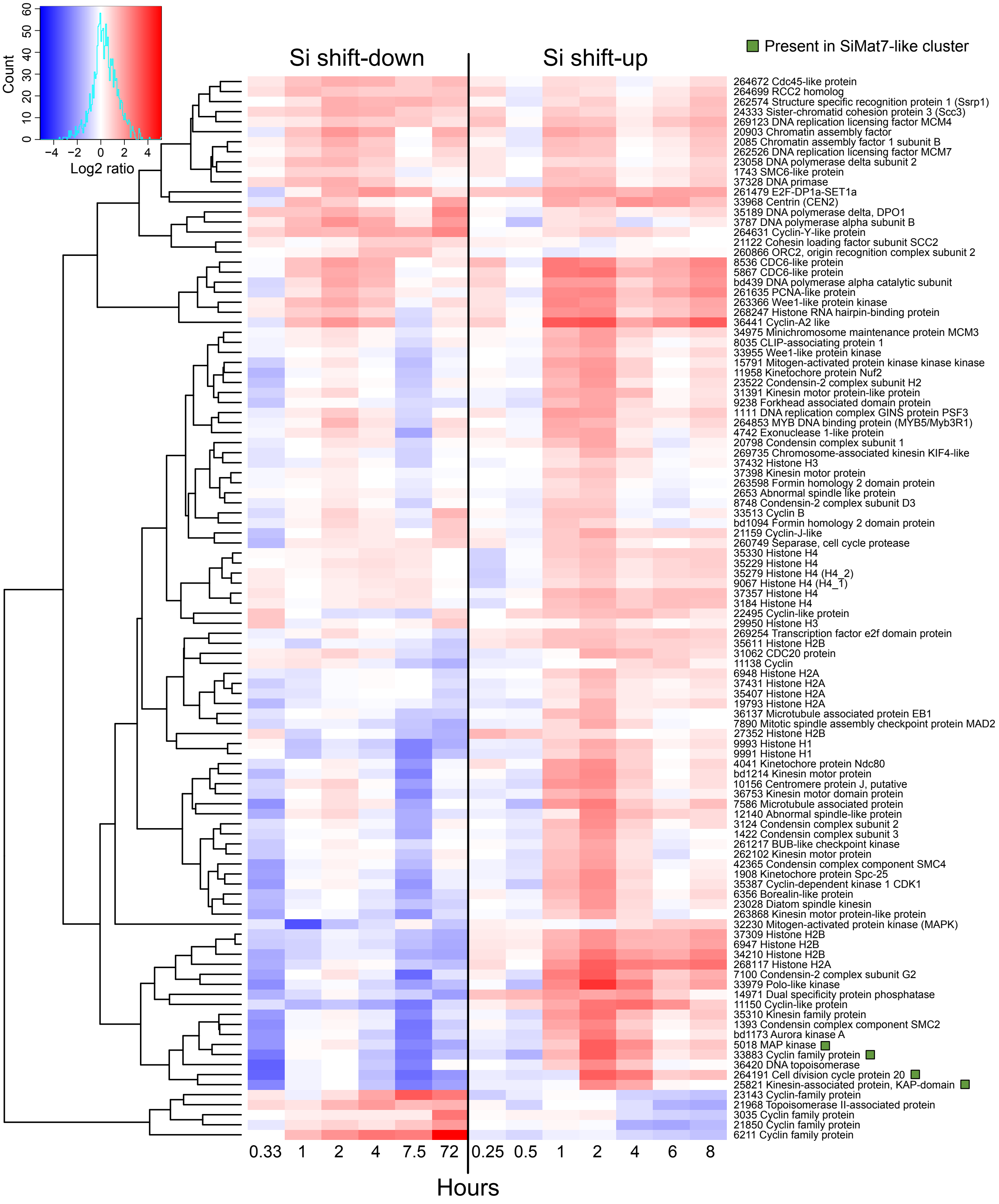

Supplementary Figure S8 Si-dependent responses of cell cycle-related genes. A cluster analysis was performed on 106 genes encoding cell cycle-related proteins. The heat map represents log2-transformed fold change in expression for the indicated time points. Numbers indicate JGI Thaps3 gene IDs. The colour key and histogram is shown to the left. The green squares indicate genes that also are present in the SiMat7-like gene cluster (Supplementary Figure S5).

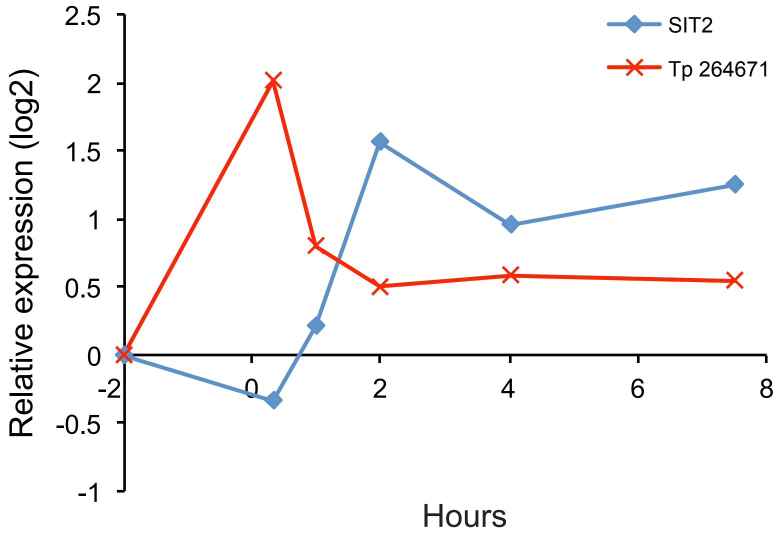

Supplementary Figure S9 The kinase *Tp264671* has similar expression to the silicon transporter *SIT2* during Si shift-down. Expression of *Tp264671* and *SIT2* represented as log2-transformed fold change during Si shift-down.

Table S1. Comparison of this study with Mock et al. (2008). The 15 genes showing highest Log2-transformed expression ratios of the Si shift-down experiment (96h) published by Mock et al. (2008) compared with the 72h time point of the Si shift-down experiment -2h time point. Controls in both experiments are f/2 + Si-grown cultures.

**GenBank Acc ID**	**Thaps3_PID**	**Description**	**Si- 96h log2 Mock et al. (2008)[a]**	**Si- 72h log2 (this study)**
XM_002290664.1	THAPSDRAFT_268895	Silicon transporter (SIT1)	7,15	5,77
XM_002293610.1	THAPSDRAFT_9619	Unknown hypothetical protein	6,95	5,50
XM_002288193.1	THAPSDRAFT_21665	Unknown diatom-specific protein	5,86	6,74
XM_002288194.1	THAPSDRAFT_3250	Unknown diatom-specific protein	5,86	6,30
XM_002296651.1	THAPSDRAFT_10363	SET-domain protein	5,43	3,08
XM_002290433.1	THAPSDRAFT_5835	Predicted protein	4,25	2,60
XM_002292599.1	THAPSDRAFT_24164	Unknown diatom-specific protein	4,09	1,60
XM_002288504.1	THAPSDRAFT_3863	DadA family oxidoreductase	3,58	2,00
XM_002289356.1	THAPSDRAFT_22332	Glutamine amidotransferase	3,46	2,12
XM_002288820.1	THAPSDRAFT_261820	Putative cyclin-dependent kinase	3,46	1,78
XM_002288912.1	THAPSDRAFT_3706	Unknown diatom-specific protein	3,32	1,58
XM_002295884.1	THAPS_41392	Silicon transporter 2 (SIT2)	3,17	1,75
XM_002296533.1	THAPSDRAFT_681	Amino acid/polyamine transporter	3,00	0,65
XM_002296191.1	THAPS_23505	Predicted protein	2,81	-0,40
XM_002293778.1	THAPSDRAFT_24918	Predicted protein	2,81	1,34

[a]**Mock T, Samanta MP, Iverson V, Berthiaume C, Robison M, Holtermann K, Durkin C, Bondurant SS, Richmond K, Rodesch M, et al. 2008.** Whole-genome expression profiling of the marine diatom *Thalassiosira pseudonana* identifies genes involved in silicon bioprocesses. *Proceedings of the National Academy of Sciences USA* **105:** 1579-1584.

Table S2. New KXXK motif-encoding genes in *T. pseudonana*.

Thaps3_PID	GenBank Acc. No	Length (AA)	Signal peptide	# KXXK repeats	Pentalysine cluster	PT repeats
THAPSDRAFT_1055	XM_002285993	497	X	2		
THAPSDRAFT_5357	XM_002290196	593	X	3	X	X
THAPSDRAFT_12193-THAPSDRAFT_12192[a]	XM_002295309 XM_002295308	566	X	3	X	X
THAPSDRAFT_12207	XM_002295268	763	X	4	X	X
THAPSDRAFT_20597	XM_002286532	487	X	2	X	
THAPSDRAFT_22220	XM_002289253	508	X	4		
THAPSDRAFT_23861	XM_002292091	214	X	4	X	
THAPSDRAFT_24383	XM_002296587	470	X	3	X	
THAPSDRAFT_25912/SiMat4	XM_002295310	534	X	3	X	X
THAPSDRAFT_25913	XM_002295266	709	X	2	X	

[a]THAPSDRAFT_12193 and THAPSDRAFT_12192 likely constitute the N- and C-terminal part of the same gene.

Table S3. Species included in the microalgal transcriptome analysis. Columns are labeled with the species included in the database, the phyla/classes represented, and the taxa corresponding to each phylum/class.

**Phylum/Class**	**Taxon**	**Species**
Coscinodiscophyceae	Attheya	Attheya septentrionalis CCMP2084
	Chaetoceros	Chaetoceros affinis CCMP159
		Chaetoceros brevis CCMP164
		Chaetoceros cf. neogracile RCC1993
	Corethron	Corethron hystrix 308
	Cyclotella	Cyclotella meneghiniana CCMP 338
	Leptocylindrus	Leptocylindrus danicus
	Proboscia	Proboscia alata PI_D3
	Skeletonema	Skeletonema costatum 1716
		Skeletonema menzelii CCMP793
	Thalassiosira	Thalassiosira antarctica CCMP982
		Thalassiosira gravida GMp14c1
		Thalassiosira miniscula CCMP1093
		Thalassiosira oceanica
		Thalassiosira pseudonana CCMP1335
		Thalassiosira rotula CCMP3096
		Thalassiosira weissflogii CCMP1010
Mediophyceae	Ditylum	Ditylum brightwellii GSO104
		Ditylum brightwellii GSO105
	Hemiaulus	Hemiaulus sinensis
	Minutocellus	Minutocellus polymorphus CCMP3303
	Odontella	Odontella aurita isolate 1302-5
	Triceratium	Triceratium dubium CCMP147
Fragilariophyceae	Asterionellopsis	Asterionellopsis glacialis CCMP134
	Ceratoneis	Ceratoneis closterium
	Cyclophora	Cyclophora tenuis ECT3854
	Grammatophora	Grammatophora oceanica CCMP 410
	Licmophora	Licmophora paradoxa CCMP2313
	Striatella	Striatella unipunctata CCMP2910
	Thalassionema	Thalassionema nitzschioides L26_B
	Thalassiothrix	Thalassiothrix antarctica L6_D1
Bacillariophycidae	Amphora	Amphora coffeaeformis CCMP127
	Craspedostauros	Craspedostauros australis CCMP3328
	Cylindrotheca	Cylindrotheca closterium KMMCCB-181
	Fragilariopsis	Fragilariopsis cylindrus CCMP1102
		Fragilariopsis kerguelensis L2-C3
	Nitzschia	Nitzschia punctata CCMP561
		Nitzschia sp. ChengR-2013
	Phaeodactylum	Phaeodactylum tricornutum CCAP 1055/1
	Pseudo-nitzschia	Pseudo-nitzschia arenysensis B593
		Pseudo-nitzschia australis 10249_10_AB
	Seminavis	Seminavis robusta 85A/85B
	Stauroneis	Stauroneis constricta CCMP1120
Bolidophyceae	Bolidomonas	Bolidomonas pacifica
Cercozoa	Lotharella	Lotharella globosa CCCM811
Choanoflagellatea	Acanthoeca-like	Acanthoeca-like sp10tr

Ciliophora	Favella	Favella taraikaensis
Cryptophyta	Goniomonas	Goniomonas pacifica CCMP1869
Chrysophyceae	Dinobryon	Dinobryon sp UTEXLB2267
	Nannochloropsis	Nannochloropsis gaditana CCMP526
		Nannochloropsis oceanica CCMP1779
	Ochromonas	Ochromonas sp CCMP1393
	Paraphysomonas	Paraphysomonas imperforata PA2
Dinoflagellata	Alexandrium	Alexandrium tamarense CCMP1771
	Karlodinium	Karlodinium micrum CCMP2283
Dictyochophyceae	Pseudopedinella	Pseudopedinella elastica CCMP716
	Pteridomonas	Pteridomonas danica PT
Euglenophyta	Eutreptiella	Eutreptiella gymnastica-like CCMP1594
Haptophyceae	Chrysochromulina	Chrysochromulina polylepis CCMP1757
	Emiliania	Emiliania huxleyi
	Isochrysis	Isochrysis galbana CCMP1323
	Pavlova	Pavlova sp CCMP459
Raphidophyceae	Chattonella	Chattonella subsalsa CCMP2191
	Heterosigma	Heterosigma akashiwo CCMP2393
		Heterosigma akashiwo CCMP452
Rhodophyta	Rhodella	Rhodella maculata CCMP736
Xanthophyceae	Vaucheria	Vaucheria litorea CCMP2940
